# BAG3 Directly Interacts with Mutated alphaB-Crystallin to Suppress Its Aggregation and Toxicity

**DOI:** 10.1371/journal.pone.0016828

**Published:** 2011-03-15

**Authors:** Akinori Hishiya, Mortada Najem Salman, Serena Carra, Harm H. Kampinga, Shinichi Takayama

**Affiliations:** 1 Cardiovascular Group, Boston Biomedical Research Institute, Watertown, Massachusetts, United States of America; 2 Department of Radiation and Stress Cell Biology, University Medical Center Groningen, University of Groningen, Groningen, The Netherlands; University of Oldenburg, Germany

## Abstract

A homozygous disruption or genetic mutation of the *bag*3 gene causes progressive myofibrillar myopathy in mouse and human skeletal and cardiac muscle disorder while mutations in the small heat shock protein αB-crystallin gene (*CRYAB*) are reported to be responsible for myofibrillar myopathy. Here, we demonstrate that BAG3 directly binds to wild-type αB-crystallin and the αB-crystallin mutant R120G, via the intermediate domain of BAG3. Peptides that inhibit this interaction in an *in vitro* binding assay indicate that two conserved Ile-Pro-Val regions of BAG3 are involved in the interaction with αB-crystallin, which is similar to results showing BAG3 binding to HspB8 and HspB6. BAG3 overexpression increased αB-crystallin R120G solubility and inhibited its intracellular aggregation in HEK293 cells. BAG3 suppressed cell death induced by αB-crystallin R120G overexpression in differentiating C2C12 mouse myoblast cells. Our findings indicate a novel function for BAG3 in inhibiting protein aggregation caused by the genetic mutation of *CRYAB* responsible for human myofibrillar myopathy.

## Introduction

Myofibrillar myopathy is a genetically heterogeneous group of diseases characterized by disrupted Z-disc structure and myofibrillar degeneration [1]. Mutations in genes encoding Z-disc components or their interacting proteins have been identified as being responsible for myofibrillar myopathy development [2], [3]. Myofibrillar myopathy is sometimes accompanied by cytosolic aggregated proteins and ectopic accumulation of various different myofibrillar proteins, suggesting that the disease may be due to abnormalities in protein folding and Z-disc protein assembly [4], [5]. αB-crystallin expression predominantly occurs in the eye lens, heart, and skeletal muscle, and point mutations in *CRYAB* cause human cataracts and myofibrillar myopathy. At least four different mutations in *CRYAB* have been detected in myofibrillar myopathy patients: R120G (substitution of Glycine for Arginine at amino acid 120), 464DelCT (a C-terminal truncation), Q151X (a C-terminal truncation), and G154S (substitution of Serine for Glycine at amino acid 154) [5], [6]. Among these, the R120G mutation has been characterized extensively [7], [8], [9], [10]. This mutation produces structural changes that result in aggregation of the protein in cells [4], [11]. The αB-crystallin R120G mutant also causes aberrant structural changes in the intermediate cytoskeletal protein desmin. Heart-specific overexpression of αB-crystallin R120G has been used to analyze the effects of this mutation on apoptosis *in vivo* [12]. Upon αB-crystallin R120G overexpression, amyloidogenic oligomers were detected in the heart, suggesting that toxic oligomers generated by this mutation may affect apoptotic pathways by inducing mitochondrial dysfunction and disrupting the cytoskeletal network [8], [12].

Protein structure is essential for proper protein function. Although the information for protein structure is conserved at the primary amino acid sequence level, many proteins are easily misfolded in the crowded cellular milieu. Protein folding systems are widely conserved across species, and play a significant role in maintaining proper protein structure in cells. The importance of protein folding is well recognized since many diseases result from abnormal protein folding. Molecular chaperones and co-chaperones are vital for correct protein folding, inhibition of protein aggregation, and degradation of misfolded proteins. There are two major cytosolic molecular chaperones: stress-inducible Hsp70 and constitutively expressed Hsc70. BAG3 is a member of the Bcl-2-associated athanogene (BAG) family of proteins that regulate Hsp70/Hsc70 chaperone activity via their conserved C-terminal domain [13]. The upstream sequences of the BAG family proteins are quite divergent, which presumably allows the different family members to act in a variety of settings. BAG3 carries both a WW domain and PXXP motif, which are well known protein interaction motifs [14], [15].

BAG3 null mutation mice show severe striated muscle degeneration and early lethality, characterized by myofibrillar myopathy [16]. Our recent research revealed the important role of BAG3 in the maintenance of myofibriller structure [17]. Furthermore, a mutation in the *bag*3 gene, that results in substitution of proline for leucine at amino acid 209 (P209L), was identified in patients having progressive-type myofibrillar myopathy [18], [19]. In addition, BAG3 protein co-localized with sites of desmin aggregation found in skeletal muscle from canine myofibrillar myopathy [20]. These data suggest that BAG3 has important roles in preventing protein aggregation and maintaining myofibrillar structure in striated muscle.

Here we report that BAG3 directly binds to wild-type αB-crystallin and R120G αB-crystallin and inhibits the aggregation and increased apoptosis induced by this mutation. This study reveals a potential connection between BAG3 and small heat shock proteins to prevent the protein aggregation and cell death that occurs in myofibrillar myopathy.

## Materials and Methods

### Antibodies

A polyclonal antibody that recognizes BAG3 was described previously [16]. Mouse monoclonal antibodies for FLAG and α-actinin (sarcomeric) were purchased from SIGMA (SIGMA, St Louis, MO) while antibodies for αB-crystallin and actin were obtained from Assay Designs, Inc. (Ann Arbor, MI) and Neomarker (Fremont, CA), respectively. All other antibodies were from Santa Cruz Biotechnology (Santa Cruz, CA).

### Plasmid Construction

The plasmid for human BAG3 was described in [16], [21]. cDNAs for human αB-crystallin wild type and its mutant (R120G) inserted into pRcCMV were a generous gift from Drs. H. Ito and K. Kato (Aichi Human Service Center). Hsc70 cDNA was amplified using RNA extracted from HEK293 cell, followed by RT-PCR and subcloned into pcDNA3 Flag vecotor (Genbank ID: NP_006588). For expression of GST fusion proteins in *E. coli* strain BL21, these cDNAs were also subcloned into pGEX-6P-1 (Amersham Biosciences, Uppsala, Sweden). Adeno-X Expression System Kit was used for adenovirus production (BD Biosciences Clontech). Adenoviral vector encoding β-galactosidase was used as a control.

### Cell culture

Cardiomyocytes were isolated from hearts of neonatal rats (Harlan, Indianapolis, IN) using the Neonatal Cardiomyocyte Isolation System (Worthington Biochemical Corporation, Lakewood, NJ). C2C12 and HEK293 cells were purchased from ATCC (Manassas, VA). These cells were cultured at 37°C in Dulbecco's modified Eagle's medium (DMEM) containing 10% FBS supplemented with penicillin and streptomycin. Transpass (New England Biolabs) or Lipofectamine 2000 (Invitrogene) was used for transfection. For differentiation of C2C12 cells, growth medium was replaced for differentiation medium (DMEM containing 2% horse serum supplemented with penicillin and streptomycin).

### Proteins

GST-BAG3 and GST-αB-crystallin were expressed in *E. coli* strain BL21. Cells were lysed in lysozyme lysis buffer (50 mM Tris, pH 8.0, 2 mM EDTA, 100 mM NaCl, 1% Triton X-100, 200 mM NaSCN, 1 mg/ml lysozyme) supplemented with a mixture of protease inhibitors (CompleteTM, Roche Diagnostics). After brief sonication, the cell lysate was centrifuged at 18,000×g for 15 min at 4°C, and the supernatant incubated with GSH-sepharose 4B (Amersham Pharmacia Biosciences) for 2 hrs. The beads were then washed four times with lysis buffer. Protein expression and purification was confirmed by immunoblot assay.

### Immunoprecipitation

For *in vivo* association, HEK293 cells were transiently transfected with various plasmids. 48 hours after transfection, cells were lysed in immunoprecipitation buffer (20 mM Tris, pH 7.5, 150 mM NaCl, 1 mM EDTA, 1 mM EGTA, 10 mM NAF, 2 mM Na_3_VO_4_, 2 mM PMSF, and 1% TritonX-100) supplemented with a mixture of protease inhibitors (CompleteTM, Roche Diagnostics). Pre-cleared lysates were subjected to immunoprecipitation with indicated antibodies. Precipitated proteins were eluted from the beads by boiling in SDS sample buffer, and separated by SDS-PAGE. Immunoblot assays were performed using the indicated antibodies.

### *In vitro* binding

To detect protein interactions *in vitro*, a GST pull-down assay was performed. GST, GST fusion αB-crystallin, and BAG3 were expressed in BL21 cells, induced by 1 mM isopropyl-1-thio-β-galactopyranoside, and purified using glutathione-Sepharose beads (Amersham Pharmacia Bioscience, Uppsala, Sweden). Purified proteins were incubated in immunoprecipitation buffer followed by precipitation with glutathione-Sepharose beads (Amersham Pharmacia Bioscience). Bound proteins were eluted from the beads by boiling in SDS sample buffer, separated by SDS-PAGE, and visualized by immunoblotting using anti-BAG3 or anti-αB-crystallin antibody.

### Cell Lysis

HEK293 cells were lysed in lysis buffer A (10 mM Tris, pH 8.0, 150 mM NaCl, 2% SDS, 10 mM NaF, 2 mM Na_3_VO_4_, 2 mM PMSF, and 1∶50 protease inhibitor cocktail), and sonicated; this sample represented the “total fraction”. Lysis buffer B (20 mM Tris, pH 7.4, 150 mM NaCl, 0.001% Tween-20, 10 mM NaF, 2 mM Na_3_VO_4_, 2 mM PMSF, and 1∶50 protease inhibitor cocktail) was used to isolate the soluble fraction. After homogenization, the sample was transferred to a tube, and centrifuged at 14,000×g for 15 minutes at 4°C. The supernatant was transferred to a new tube to represent the “soluble fraction”.

### Immunofluorescence

For staining of cultured cells, cells were washed with PBS, and fixed with 4% paraformaldehyde in PBS for 5 minutes. After three 5-min washes with PBS, the fixed samples were incubated in 0.2% Triton X-100 in PBS for 3 minutes and blocked with 2% BSA in PBS for 1 hour. Various primary antibodies used for staining were incubated with the samples for 1 hour at room temperature. After washing with PBS, the samples were incubated with Alexa Fluor conjugated antibodies (Alexa Fluor 488 goat anti-mouse IgG and Alexa Fluor 594 goat anti-rabbit IgG (Molecular Probes, Eugene, OR)). After washing with PBS, samples were mounted using Vectashield with Dapi (4′, 6-diamidino-2-phenylindole: Vector Laboratories, Burlingame, CA). For apoptosis assays, cells were stained with αB-crystallin and DAPI, and cells containing nuclear fragmentation were counted.

### Immunoblotting

Extracted proteins were subjected to SDS-polyacrylamide gel electrophoresis (SDS-PAGE), transferred onto polyvinylidene difluoride membranes (Amersham Pharmacia Biosciences), and probed with the indicated antibodies. Signals were visualized using ECL Plus reagents (Amersham Pharmacia Biosciences). Protein concentrations were measured using the BCL protein assay reagent (PIERCE, Rockford, IL).

### Statistics

Data are expressed as mean ± S.E. A paired Student's t-test was used to analyze differences between two groups, and p vales of <0.05 or <0.01 were considered significant. “n.s” stands for not significant. Image J is a public domain, Java-based image processing program developed at the National Institutes of Health (NIH).

## Results

### BAG3 interacts with αB-crystallin through the BAG3 intermediate domain

**Figure 1:**
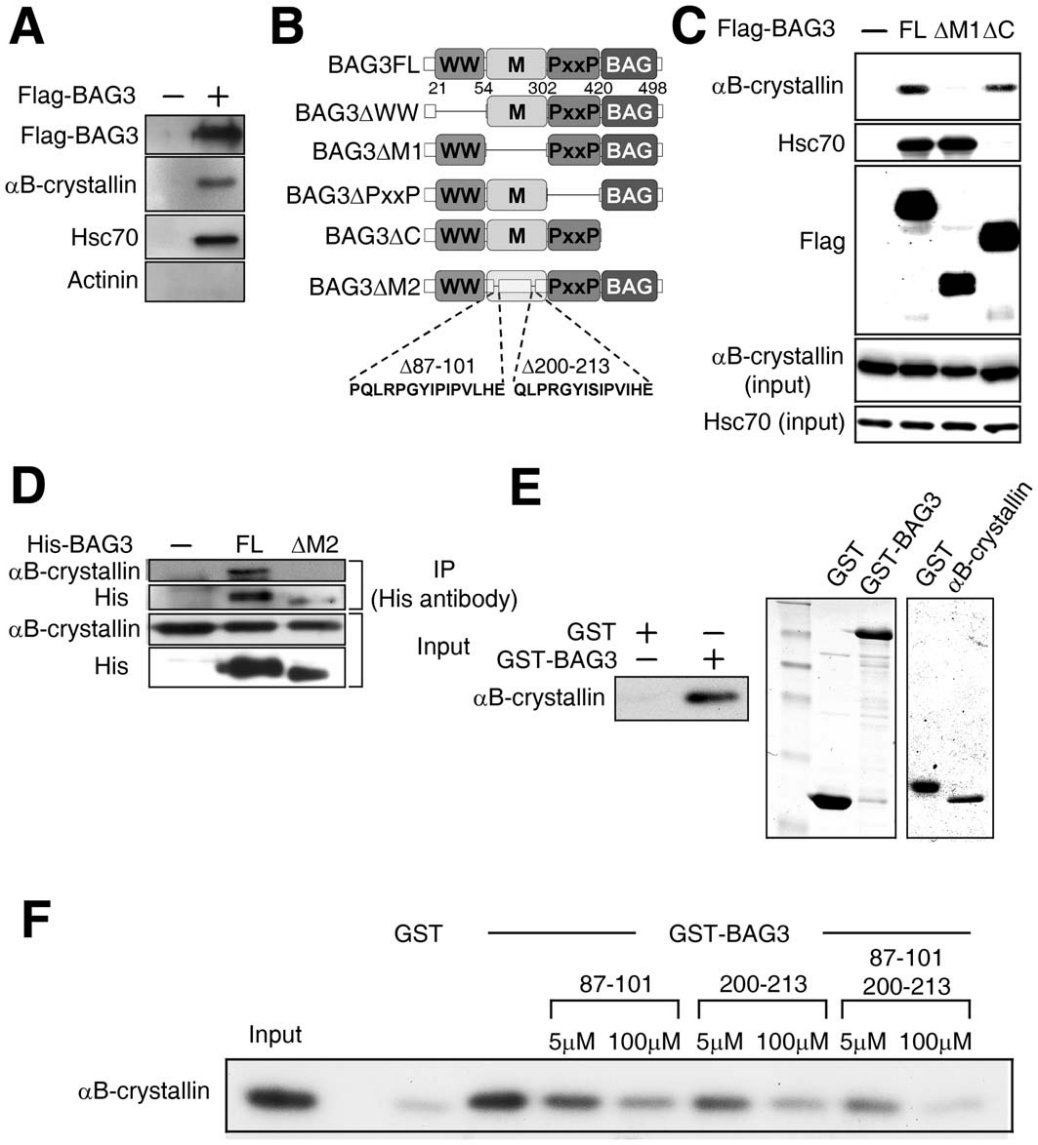
BAG3 binds αB-crystallin directly through BAG3 intermediate region. (A) *BAG3 interacts with αB-crystallin in rat cardiomyocytes*. Cardiomyocytes were isolated from rat hearts, and Flag-tagged BAG3 was expressed using adenovirus. Cell extracts were immunoprecipitated with anti-Flag antibody, and the sample was applied to SDS-PAGE. Anti-αB-crystallin antibody was used to detect αB-crystallin. The same membrane was blotted with anti-Hsc70 and anti-αActinin antibody for a positive and negative control, respectively. No actinin interacting was observed (negative control). (B) *Schematic representation of the primary structure of wild-type BAG3 and its mutants*. The BAG3ΔC construct lacks the BAG domain. The BAG3 intermediate domain and amino acids from 87–101 and from 200 to 213 are deleted in BAG3ΔM1 and BAG3ΔM2, respectively. Amino acid sequences between residues 87–101 and 200–213 are shown. (C) *Co-precipitation of αB-crystallin and BAG3*. Flag-tagged BAG3 or mutants were expressed with αB-crystallin in HEK293 cells, and cell extracts were immunoprecipitated with anti-Flag antibody. αB-crystallin was detected with anti-αB-crystallin antibody. Anti-Hsc70 antibody was used to detect Hsc70 precipitation with BAG3. The cell extracts were also applied to an immunoblotting assay to verify expression. (D) *BAG3 interacts with αB-crystallin via amino acids 87–101 and 200–213*. His-tagged BAG3 or BAG3M2 was expressed with αB-crystallin in HEK293 cells, and BAG3 was precipitated with anti-His antibody. Co-precipitated αB-crystallin was detected with anti-αB-crystallin antibody (upper panel). The same membrane was blotted with anti-His antibody (bottom panel). (E) *Direct interaction of BAG3 with αB-crystallin*. Left panel: GST protein fused to BAG3 was expressed in *Escherichia coli*, and purified fusion proteins were incubated with purified αB-crystallin, followed by precipitation with GSH beads. After SDS-PAGE, αB-crystallin was detected using anti-αB-crystallin antibody. Right panel: GST fusion proteins used in the experiment were visualized with CBB staining to verify the quality and quantity. *(F) Competition assay with peptides*. Purified GST or GST-BAG3 was incubated with purified αB-crystallin in the presence or absence of peptides corresponding to amino acids 87–101 or 200–213 of BAG3 at indicated concentrations. The precipitated samples with GSH beads were loaded onto SDS-PAGE, followed by an immunoblotting assay using anti-αB-crystallin antibody.

BAG3 is highly expressed in striated muscle and null mutations in BAG3 are implicated in myofibrillar myopathy [16]. The small heat shock protein (sHsp), αB-crystallin, is also expressed in striated muscle at relatively high levels and a mutation in this protein (R120G) is also reported to cause myofibrillar myopathy [4]. Thus, we investigated whether BAG3 and αB-crystallin are associated in cultured striated muscle, in this case, rat neonatal cardiomyocytes. Flag-tagged BAG3 was expressed in rat neonatal cardiomyocytes using adenovirus, and an immunoprecipitation assay was performed. Immunocomplexes were prepared using anti-Flag antibody, followed by SDS-PAGE to detect endogenous αB-crystallin. The results showed that BAG3 bound to αB-crystallin as well as Hsc70 (Figure 1A). To identify the binding region required for this interaction, we expressed various BAG3 mutants in HEK293 cells with αB-crystallin, and performed immunoprecipitation assays. The BAG3 protein possesses three known protein interaction motifs: the WW domain, which interacts with the PPXY sequence found in various subcellular compartments [14]; the PxxP motif, a candidate interaction site for SH3 domain proteins [22]; and the BAG domain, which interacts with Hsp70/Hsc70. We also constructed a BAG3 mutant (ΔM1) that lacks the region between the WW domain and PxxP motif (Figure 1B). While wild-type BAG3 interacted with both αB-crystallin and Hsc70, the ΔM1 mutant failed to associate only with αB-crystallin, while the ΔBAG mutant did not interact with Hsc70 (Figure 1C), suggesting that BAG3 binds to both αB-crystallin and Hsc70 via two distinct regions. Recently, Margit *et al.* explored the precise region of BAG3 involved in binding with other sHsps, and found that HspB8 and HspB6 required BAG3 amino acid residues 87–101 and 200–213, respectively [21]. Since this region is located in the BAG3 intermediate domain, we verified whether αB-crystallin also binds to BAG3 at the same region. We expressed in HEK293 cells αB-crystallin along with His-BAG3 wild type or its mutant (ΔM2: deletion that includes both aa 87–101 and aa 200–213), which lacks the interacting domain for HspB8/HspB6, and the cell extract was incubated with anti-His antibody to isolate His-tagged proteins. As expected, the interaction of BAG3 with αB-crystallin was abolished when the BAG3ΔM2 mutant was expressed instead of wild type BAG3 (Figure 1D). The direct interaction between BAG3 and αB-crystallin was also confirmed by an *in vitro* binding assay using purified proteins (Figure 1E). We generated two peptides corresponding to aa 87–101 and aa 200–213 of BAG3, and performed a competition assay using purified BAG3 and αB-crystallin. Each peptide partially hampered the interaction between BAG3 and αB-crystallin, and the interaction was completely abolished when both peptides were present (Figure 1F).

### BAG3 interacts with αB-crystallin R120G

**Figure 2:**
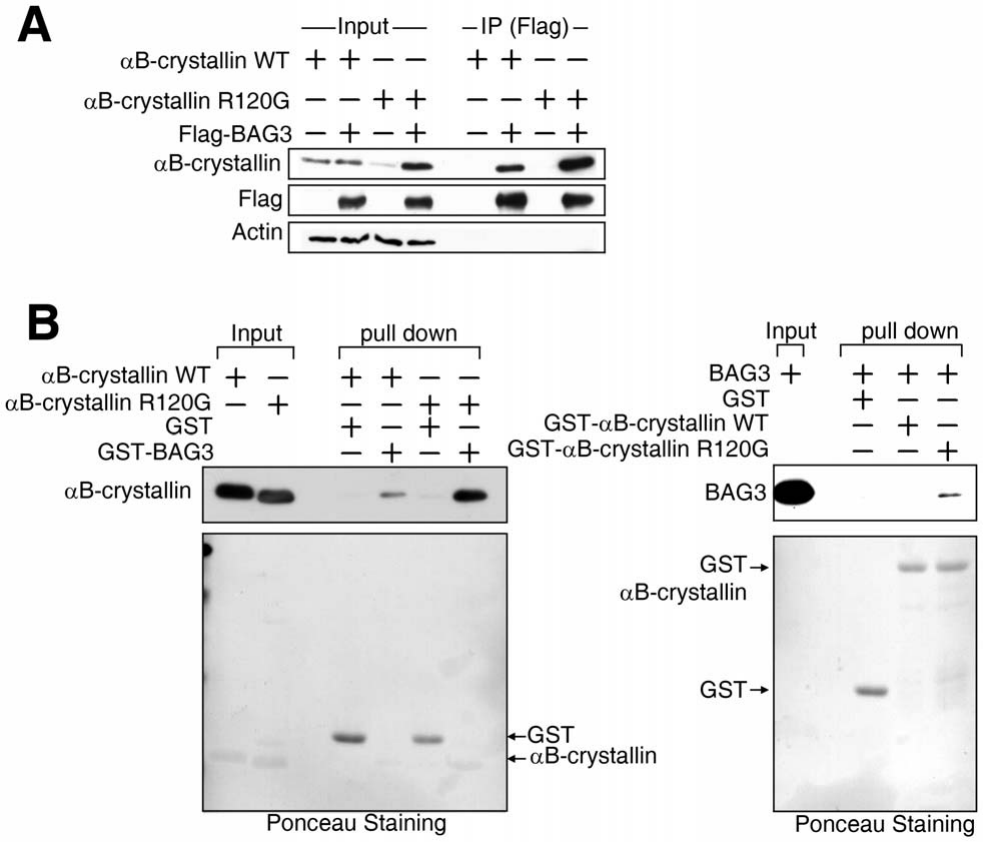
BAG3 recognizes folding status of αB-crystallin. *(A) BAG3 binds to mutant type of αB-crystallin strongly.* Wild type (WT) or mutant (R120G) αB-crystallin was expressed in HEK293 cells with Flag-tagged BAG3, and the cell lysate was mixed with anti-Flag antibody. Precipitated samples were analyzed by SDS-PAGE using anti-αB-crystallin (upper panel), Flag-tagged BAG3 (middle panel), and actin (lower panel). The sample before immunoprecipitation was also loaded to confirm protein expression (right four lanes). *(B) Direct recognition of mutant αB-crystallin by BAG3.* Purified GST or GST-fused BAG3 beads were incubated with purified αB-crystallin wild type (WT) or mutant (R120G), and a pull-down assay was performed. Precipitated αB-crystallin was detected with anti-αB-crystallin antibody (upper panel). The same membrane stained with Ponceau is shown below. *Mutated αB-crystallin preferentially binds to BAG3.* Purified GST, GST-fused αB-crystallin wild type (WT) or mutant (R120G) was mixed with purified BAG3 for a pull-down assay. The detection of BAG3 was achieved with anti-BAG3 antibody after SDS-PAGE. The same membrane was stained with Ponceau (lower panel).

The previous experiments indicate a direct interaction between BAG3 and αB-crystallin. To confirm whether BAG3 also interacts with αB-crystallin mutants in cells, either wild-type or αB-crystallin R120G was expressed with Flag-tagged BAG3 in HEK293 cells, and an immunoprecipitation assay was performed using anti-Flag antibody. αB-crystallin R120G alone was expressed at lower levels compared to wild-type, but the band intensity increased noticeably when BAG3 was co-expressed (Figure 2A). Since BAG3 overexpression with wild type αB-crystallin did not show increased signals, the interaction of BAG3 with αB-crystallin R120G may increase the stability of R120G αB-crystallin perhaps due to conformational changes [23]. In this assay, we used immunoprecipitation buffer (Materials and Methods) for cell lysis. The difference in αB-crystallin signal in Figure 2A suggests that αB-crystallin R120G be in the insoluble fraction in the absence of BAG3 co-expression, but in the presence of BAG3 overexpression, the solubility of αB-crystallin R120G is enhanced. To investigate whether BAG3 recognizes R120G αB-crystallin directly, we purified wild type αB-crystallin and αB-crystallin R120G from *E.coli*, and mixed the proteins with GST or GST-BAG3 in a pull-down assay using Glutathione-sepharose beads. Increased amounts of αB-crystallin R120G precipitated with BAG3 relative to wild type αB-crystallin (Figure 2B). The same result was obtained using purified BAG3 mixed with GST-αB-crystallin, followed by pulled down with glutathione sepharose beads (Figure 2C).

### BAG3 reduces aggregation and increases solubility of αB-crystallin mutant

**Figure 3:**
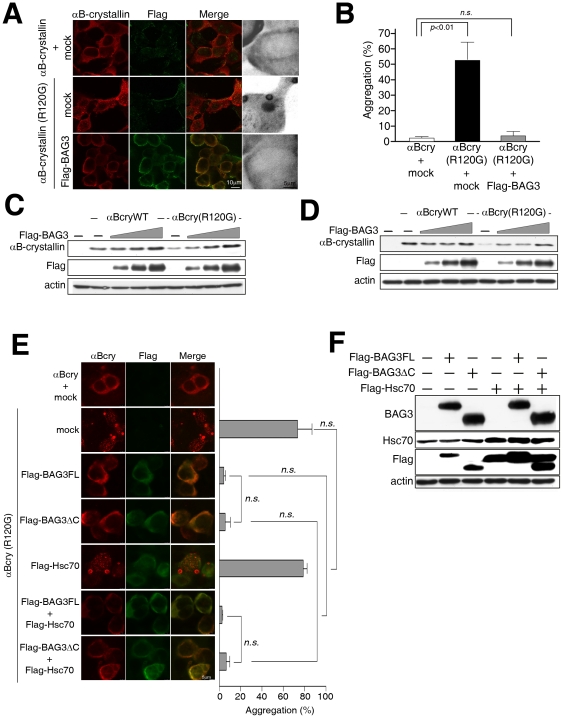
Overexpression of BAG3 suppresses aggregation of mutant αB-crystallin and increases its solubility. (A) *Aggregation of mutant αB-crystallin is cleared by BAG3.* HEK293 cells were transfected with plasmids for wild type or mutant αB-crystallin with or without Flag-tagged BAG3. Two days later, cells were fixed and αB-crystallin and Flag-tagged BAG3 were stained with anti-αB-crystallin antibody (red in color images) and Flag-antibody (green in color images), respectively. Merged images are also shown (Merge). (B) *BAG3 cleared aggregation caused by αB-crystallin mutant.* After staining of αB-crystallin, the number of cells containing αB-crystallin aggregation were counted and statistically analyzed. The Y-axis indicates the percentage of cells displaying αB-crystallin aggregation. *(C) BAG3 increases stability of mutant type of αB-crystallin in HEK293 cells.* HEK293 cells were cultured in a 24-well dish and plasmids for αB-crystallin wild type (WT) or mutant (R120G) were transfected (0.2 µg each) with or without the plasmid for Flag-tagged BAG3. The amount of BAG3 plasmid was increased gradually (0.05, 0.15, and 0.3 µg). After two days, cells were lysed in lysis buffer A (10 mM Tris, pH 8.0, 150 mM NaCl, 2% SDS, 10 mM NaF, 2 mM Na_3_VO_4_, 2 mM PMSF, and 1∶50 protease inhibitor cocktail), and sonicated. The sample was loaded onto SDS-PAGE, and an immunoblotting assay performed with anti-αB-crystallin (first panel), Flag (second panel), and actin antibody (third panel). GFP plasmid was transfected together to monitor transfection efficiency, and detected with anti-GFP antibody (bottom panel). *(D) BAG3 increases solubility of mutant type of αB-crystallin in HEK293 cells.* The same set of transfections described above in “C” were done, but lysis buffer B was used instead of lysis buffer A (lysis buffer B; 20 mM Tris, pH 7.4, 150 mM NaCl, 0.001% Tween-20, 10 mM NaF, 2 mM Na_3_VO_4_, 2 mM PMSF, and 1∶50 protease inhibitor cocktail). After homogenization, the sample was transferred to a tube, and centrifuged at 14,000×g for 15 minutes at 4°C. The supernatant was transferred to a new tube to represent the soluble fraction. The samples were subjected to an immunoblotting assay to detect αB-crystallin (first panel), Flag-tagged BAG3 (second panel), actin (third panel), and GFP (last panel). (E) *BAG3 clears aggregation of mutant αB-crystallin independent with Hsc70.* HEK293 cells were transfected with indicated plasmids, and cultured for two days. Panel #1 (upper three panel): wild type αB-crystallin+pcDNA3 empty vector (mock). Panel #2–7 represents transfectants of αB-crystallin R120G mutant with plasmid indicated at the left of each three panels. After fixation, αB-crystallin (αBCry) and Flag-tagged proteins were stained with anti-αB-crystallin antibody (red in color images) and Flag-antibody (green in color images), respectively. Merged images are also shown (Merge). The number of cells containing αB-crystallin R120G aggregation were counted and statistically analyzed. The percentage of cells displaying αB-crystallin aggregation is shown in the right panel. (F) *Expression of Hsc70 and BAG3 proteins in HEK293 cells used above.* HEK293 cells were transfected with plasmids for αB-crystallin with or without Flag-BAG3 or Flag-Hsc70. Two days later, cells were lysed for western blotting. The expression levels of Hsc70 and BAG3 proteins in HEK293 cells were verified using anti-Hsc70 antibody (Hsc70), anti-BAG3 antibody (BAG3), and anti-Flag antibody (Flag). Actin was used as a loading control (actin). Hsc70 antibody detected endogenous Hsc70 as shown at the left three lanes in the second panel and overexpressed Flag tagged Hsc70 with endogenous protein at the right three lanes. Flag antibody detected BAG3FL (2 and 5 lane), BAG3▵C (3 and 6 lane) and Hsc70 (4, 5 and 6 lane).

Since BAG3 binds to both wild-type and R120G αB-crystallin, we investigated whether BAG3 overexpression affects the aggregation seen for R120G αB-crystallin. When wild type αB-crystallin was expressed in HEK293 cells, αB-crystallin had a diffuse cytosolic localization (Figure 3A). In contrast, αB-crystallin R120G expression in HEK293 cells produced a fine granular pattern with significant perinuclear aggregation (Figure 3A). To examine the effect of BAG3 overexpression on αB-crystallin aggregation, HEK293 cells were co-transfected with Flag-tagged-BAG3 and αB-crystallin R120G expression vectors. Immunocytochemical analyses were performed using anti-Flag and -αB-crystallin antibodies. In the presence of BAG3 expression, the aggregation that was observed for nearly half the cells expressing αB-crystallin R120G disappeared (Figure 3A). The yellow staining seen in the merged image of cells overexpressing Flag BAG3 and αB-crystallin R120G (right bottom) indicates that BAG3 and αB-crystallin are colocalized (right bottom). We used αB-crystallin antibody to detect overexpressed protein, due to the low levels of endogenous αB-crystallin in HEK293 with non-tagged construct of αB-crystallin expression vector (Figure 3C). To perform statistical analysis of aggregation, we calculated the number of cells where aggregation was present and absent. As shown in Figure 3B, αB-crystallin R120G overexpression induces aggregates in more than half the cells. Upon overexpression of BAG3, the number of cells with aggregation was dramatically reduced, indicating that BAG3 inhibits aggregation of mutant αB-crystallin (Figure 3B).

To investigate whether BAG3 enhances the solubility of αB-crystallin R120G, a western blot analysis was performed using two different lysis buffers, which either did (containing 2% SDS) or did not (containing 0.001% Triton X-100) solubilize protein aggregates. As shown in Figure 3C, the signal of wild type αB-crystallin (lane 2) has a similar intensity as R120G αB-crystallin (lane 6). With overexpression of BAG3, a higher intensity signal for αB-crystallin (lane 3, 4, 5) and R120G αB-crystallin (lane 7, 8, 9) was detected (Figure 3C upper panel) [23]. In contrast, using buffer with 0.001% Triton, which does not solubilize aggregates, R120G αB-crystallin was mostly in the insoluble fraction (Figure 3D lane 6). Overepxression of BAG3 increased the intensity of R120G αB-crystallin signal even with non-solubilizing 0.001% Triton buffer, indicating that BAG3 increased the solubility of R120G αB-crystallin. This could also explain the higher intensity for R120G αB-crystallin seen when BAG3 is co-expressed in Figure 2A.

To determine the effect of Hsc70/Hsp70 interaction in prevention of R120G aggregation, we used a BAG3 deletion mutant of the BAG domain (BAG3ΔC) and overexpression of Hsc70 in the aggregation assay. The expression vectors, pcDNA3 BAG3ΔC, pcDNA3 wild type BAG3 (BAG3FL), pcDNA3 empty plasmids (mock) and pcDNA3 Flag-Hsc70, were transfected with αB-crystallin R120G expression vector, followed by immunohistomical analysis and statistical analysis of aggregation using anti-αB-crystallin antibody described above (Figure 3A and B). Figure 3E shows the effect of BAG3 with or without the BAG domain and overexpression of Hsc70 in aggregation caused by the R120G mutant using immunohistochemical assay. Deletion of the BAG domain in BAG3 (BAG3ΔC) still induced a significant reduction in aggregation formation. Overexpression of Hsc70 with BAG3 (BAG3FL+Hsc70) did not cause a significant further reduction of R120G aggregation compared with BAG3ΔC+Hsc70. From these results, it appears that the effect of BAG3 in prevention of aggregation caused by R120G mutant does not depend on its interaction with Hsp70/Hsc70.

### BAG3 reduces cell toxicity caused by αB-crystallin mutation

**Figure 4:**
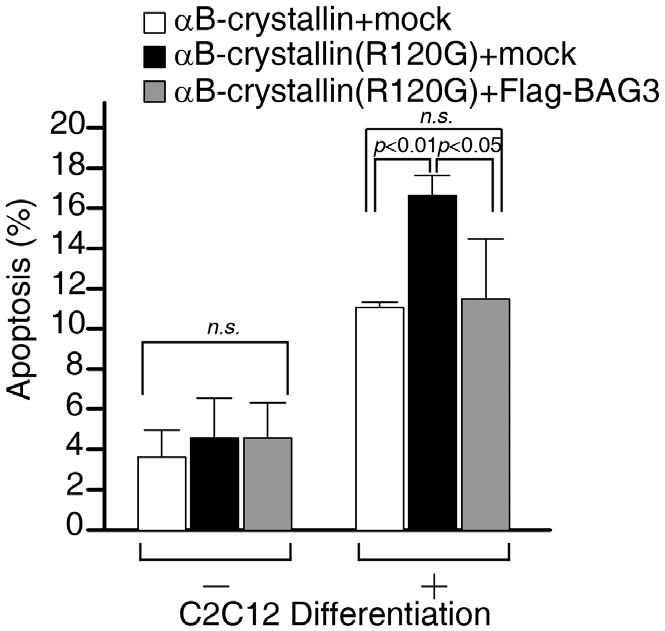
BAG3 attenuates the toxicity of mutant αB-crystallin. *BAG3 inhibits apoptosis caused by αB-crystallin mutant in C2C12 cells*. *α*B-crystallin wild type or R120G was expressed with or without Flag-tagged BAG3 in C2C12 myoblast cells, and cells were cultured with differentiation media to induce myotube differentiation. After fixing cells, αB-crystallin and cell nuclei were stained with anti-αB-crystallin antibody and Dapi solution, respectively. Cells with nuclear fragmentation typical of apoptosis were counted and statistically analyzed.

R120G αB-crystallin is reportedly toxic to cells, and induces cell death in striated muscle [8], [10], [24], [25]. To investigate whether the toxicity of R120G αB-crystallin is also inhibited by BAG3 overexpression, R120G αB-crystallin was expressed with or without Flag-tagged BAG3 in the C2C12 mouse myoblast cell line, and the number of apoptotic cells during myoblast differentiation counted. There was no obvious difference in the apoptotic cell number among cells expressing wild type αB-crystallin, mutant αB-crystallin and mutant αB-crystallin together with BAG3 in C2C12 myoblasts. Upon differentiation initiation, more apoptosis was observed for R120G αB-crystallin expressing cells. However, the number of apoptotic cells decreased when BAG3 was co-expressed with R120G αB-crystallin (Figure 4).

## Discussion

During investigations on myofibrillar myopathy caused by ablation of the *bag3* gene, we identified a direct interaction between αB-crystallin and BAG3. This interaction can inhibit the aggregation and toxicity caused by a point mutation in αB-crystallin, R120G αB-crystallin [4]. Hsp70 and small heat shock protein family molecular chaperones have been reported to be critical players in human degenerative diseases [26]. Interestingly, genetic mutations of small heat shock proteins are found in diseases involving neurons, muscular tissues and ocular lens that arise from defects in protein folding. Four mutations (R120G, 464delCT, Q151X and G154S) in the αB-crystallin gene were identified in inherited myofibrillar myopathy [4], [5], [6]. Genetic mutations in the intermediate filament protein desmin also cause mis-assembly and aggregation of desmin, resulting in myofibrillar myopathy having similar pathological features [7]. Thus, the desmin aggregation occurring in myofibrillar myopathy attributable to mutations in αB-crystallin is due to the loss of its chaperone function [11], [27], [28]. Homozygous null mutation of desmin or *CRYAB* only induces very mild myopathy compared to myopathy induced by mutated αB-crystallin (R120G), indicating that the toxicity may be induced by aggregation itself [7]. This line of thinking is supported by the ability of R120G αB-crystallin to generate pathogenic aggregation and toxic amyloidgenic oligomers both *in vivo* and *in vitro* [12]. Since αB-crystallin has chaperone activity and interacts with other chaperones, this aggregation of mutant αB-crystallin may be inhibited by different molecular chaperones. αB-crystallin R120G aggregation is reversed by overexpression of Hsp27, suggesting that Hsp27 can be a molecular chaperone for R120G αB-crystallin [29]. αB-crystallin R120G aggregation is also inhibited by wild type αB-crystallin, Hsp27 and HspB8 [30]. In addition, Hsp70 and its co-chaperone CHIP inhibit aggregation of αB-crystallin R120G [30]. Since BAG3 is a co-chaperone of Hsp70/Hsc70 and an interacting partner of HspB8, inhibition of αB-crystallin R120G aggregation can also be modified by interaction with HspB8 or Hsp70. Multiple protein complexes with BAG3, wild type αB-crystallin, HspB8 and Hsp70 may cooperatively inhibit protein aggregation generated by mutations in αB-crystallin. As shown in Figure 3E, BAG3 with deletion of the BAG domain still inhibits aggregation and apoptosis induced by the αB-crystallin R120G mutant, suggesting that BAG3 may not use Hsp70/Hsc70 for inhibiting aggregation in our system. In addition, overexpression of Hsc70 produced no additional effect in the presence of the BAG3 and BAG3 mutant. This data indicated that Hsc70 might not work synergistically with BAG3 in preventing aggregation. However, since Hsp70 and small Hsp chaperone systems have similar effects on R120G aggregation and BAG3 binds to both chaperones, it is possible that small heat shock protein interaction with BAG3 is enough to prevent aggregation in our assay system. Hsp70 inhibits R120G aggregation with ubiquitin ligase CHIP or co-chaperone Hdj1, suggesting that a particular complex with co-chaperones might be critical for Hsp70 to inhibit R120G aggregation. Thus, further investigation is necessary to elucidate the functional connection of BAG3 with Hsp70 complexes in prevention of R120G aggregation.

A BAG3 null mutation in mice results in severe progressive myofibrillar myopathy [16]. Recently, a genetic mutation where Proline is mutated to Leucine at amino acid position 209 of BAG3 has been reported in patients having severe myofibrillar myopathy [18], [19]. Interestingly, amino acid 209 is located in the region of BAG3 that interacts with small heat shock proteins (HspB6, B8 [21] and αB-crystallin Figure 1F). Recently, the relationship between molecular chaperones and autophagy has been extensively studied. HspB8, a small heat-shock protein, forms a stable complex with BAG3, and this complex accelerates the degradation of mutant Huntingtin protein through autophagy [21], [23]. We evaluated autophagy in our system, but found no evidence of accelerating autophagy in preventing aggregation of R120G αB-crystallin (not shown).

In Figure 2, pull down experiments indicated that BAG3 appears to have a greater affinity for R120G αB-crystallin relative to wild type. Since αB-crystallin forms multimers/dimers, it is possible that the molar ratio may be altered in the R120G αB-crystallin and BAG3 interaction. However, using a GST fusion of αB-crystallin for pull down experiments with BAG3 (Figure 2B right), only GST R120GαB-crystallin showed higher signals upon BAG3 precipitation, suggesting that R120G αB-crystallin may have a greater affinity for BAG3 compared to wild-type. This result is in agreement with finding that HspB8 interacts with αB-crystallin R120G to a greater extent than wild-type αB-crystallin, suggesting that this chaperone is recognizing a misfolded version of αB-crystallin [30]. Further experiments will be necessary to elucidate in detail why BAG3 appears to have a preference for αB-crystallin R120G.

BAG family proteins have a conserved Hsc70-interacting domain at their C-termini and regulate protein-folding activities [13], [31]. On the other hand, the N-terminal sequences of BAG family proteins are different and have distinct functions [16], [32], [33]. BAG1 possesses a Ubl domain (Ubiquitin-like domain) that binds to the 26S proteasome, suggesting a function for this protein in protein folding and degradation. Involvement of BAG1 in neurodegenerative disease has been documented for Alzheimer's and Huntington's disease as well as Amyotrophic lateral sclerosis [34], [35], [36]. In this report, we found that BAG3 directly binds to and enhances the solubility of mutant αB-crystallin R120G and inhibits its aggregation. Finally, the toxicity caused by αB-crystallin was attenuated by overexpression of BAG3. BAG3 is the first co-chaperone molecule that bridges two major molecular chaperones, Hsc70/Hsp70, and small heat shock proteins (at least HspB6, B8 and αB-crystallin). We anticipate that investigation of BAG3 and the two major chaperone interactions (small heat shock proteins and Hsp70/Hsc70) will reveal potential therapeutic approaches for myofibrillar myopathy and other aggregation/degenerative diseases.
